# EGFR blocker lapatinib inhibits the synthesis of matrix metalloproteinases from synovial fibroblasts

**DOI:** 10.55730/1300-0144.5442

**Published:** 2022-02-27

**Authors:** Demet YALÇIN KEHRİBAR, Hakan EMMUNGİL, Neşe BAŞAK TÜRKMEN, Osman ÇİFTÇİ, Emine SALVA, Metin ÖZGEN

**Affiliations:** 1 Department of Internal Medicine, Faculty of Medicine, Ondokuz Mayıs University, Samsun, Turkey; 2 Division of Rheumatology, Department of Internal Medicine, Faculty of Medicine, Trakya University, Edirne, Turkey; 3 Department of Pharmaceutical Toxicology, Faculty of Pharmacy, İnönü University, Malatya, Turkey; 4 Department of Pharmacology, Faculty of Medicine, Pamukkale University, Denizli, Turkey; 5 Division of Rheumatology, Department of Internal Medicine, Faculty of Medicine, Ondokuz Mayıs University, Samsun, Turkey

**Keywords:** Epidermal growth factor, lapatinib, matrix metalloproteinase, rheumatoid arthritis, synovial fibroblast

## Abstract

**Background/aim:**

Epidermal growth factor receptor (EGFR) family members and their associated ligands may be related to bone and joint destruction in rheumatoid arthritis. Matrix metalloproteinases are responsible for joint and bone tissue degradation. This study is intended to investigate the effect of epidermal growth factor receptor inhibition by lapatinib on the synthesis of matrix metalloproteinases in in vitro.

**Materials and methods:**

Synovial fibroblast cell culture was obtained from a patient with rheumatoid arthritis who underwent knee arthroplasty. Interleukin-1β (IL-1β) and tumor necrosis factor-α (TNF-α) were added to the cell culture to stimulate synovial fibroblast cells and create an inflammatory character. Understimulated and nonstimulated conditions, lapatinib was applied to the culture in four different concentrations of 25, 50, 100, and 200 μmol. Then, matrix metalloproteinase -1, -3, and, -13 levels were assessed.

**Results:**

When stimulated with IL-1β and TNF-α, the synthesis of matrix metalloproteinases from synovial fibroblast was increased significantly. When lapatinib is added to the stimulated synovial fibroblasts, matrix metalloproteinases synthesis is significantly suppressed.

**Conclusion:**

Inhibition of the EGFR pathway with lapatinib suppresses matrix metalloproteinases synthesis. Our results suggest EGFR pathway inhibition may be a promising option to prevent joint destruction in the treatment of rheumatoid arthritis.

## 1. Introduction

Rheumatoid arthritis (RA) is a disease of unknown etiopathogenesis characterized by chronic synovial inflammation, bone erosions, cartilage destruction, and pannus formation. Angiogenesis and synovial hyperplasia have an important role in the pathogenesis of the deformities that develop in RA [1–4].

Angiogenesis, one of the earliest lesions of RA, leads to hyperplastic synovium, causing progressive destruction in tendons, joint cartilage, and bone [2–4]. Rheumatoid synovium, proinflammatory mediators, cathepsin, and inflammatory cells secreting matrix metalloproteinases (MMP) play a role in this destruction, and key effector cells are synovial fibroblasts [4,5]. Epidermal growth factor (EGF) contributes to the formation of this proinflammatory and destructive setting in the joint [6]. EGF receptors are essential in the growth of hyperplastic tumors and angiogenesis. Similarly, these receptors and their ligands may be associated with synovial hyperplasia, angiogenesis, and disease progression in RA. RA synovium is also hyperplastic and invasive, and synovial fibroblasts commonly express EGF receptors (EGFRs) and their ligands [3]. Moreover, the increased serum and joint EGF and EGFR in patients with RA compared with those in the healthy population have been demonstrated by several studies [7–10]. Recently, another study provided the first evidence that EGFR polymorphisms are associated with the risk of RA development, suggesting that genetic polymorphisms in EGFR may contribute to the development of RA [11].

EGFR blockade directly leads to significant antiangiogenic effects and indirectly suppresses the synthesis of other factors with strong angiogenic effects [12–13]. In addition, EGF has been shown to stimulate the synthesis of matrix metalloproteinase, which is known to play an important role in joint destruction [7].

This data suggests that EGFR inhibition may prevent joint destruction in RA. Lapatinib, a tyrosine kinase inhibitor, is a reversible inhibitor of orally used EGFR and human EGFR-2-related tyrosine kinases [6]. This study aimed to investigate in vitro the effect of lapatinib in the synthesis of MMP from synovial fibroblast, which is key effector cells, mediating persistent inflammation, and destruction of joints.

## 2. Materials and methods

**Figure 1:**
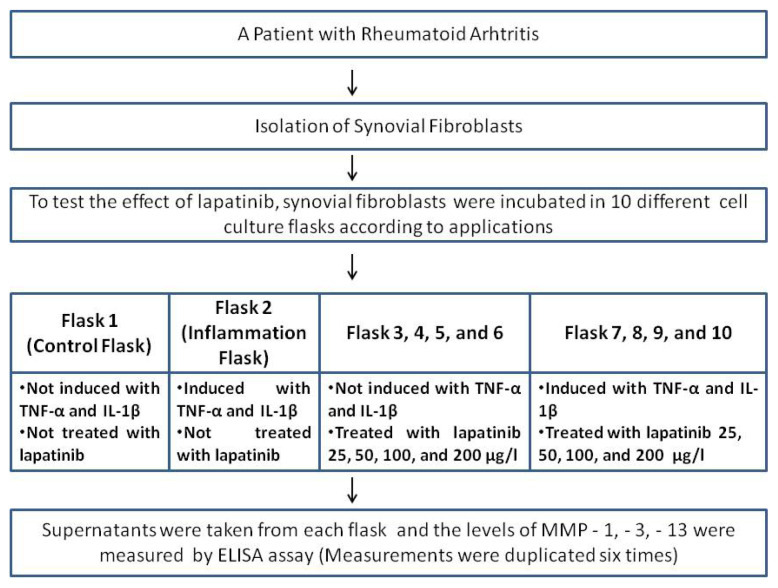
Study design.

In this study, the effect of lapatinib, an EGFR tyrosine kinase inhibitor, was investigated in vitro. Synovial fibroblasts are planned to be isolated by taking cartilage tissue from a patient diagnosed with RA (The patient with a seropositive RA diagnosis for 15 years, had not received DMARD treatment before except for methotrexate, the methotrexate treatment was discontinued 1 month before the operation, there was no comorbidity). Isolated synovial fibroblasts were inoculated on the appropriate cell culture flask. For synovial fibroblasts to take gain an inflammatory character, it was planned to add TNF-α and IL-1β. To test the effect of lapatinib, synovial fibroblasts were seeded in 10 different cell culture flasks; Flask 1; Control Flask (not induced with TNF-α and IL-1β, not treated with lapatinib), Flask 2; Inflammation Flask (induced with TNF-α and IL-1β, but not treated with lapatinib), Flask 3., 5., 7., and 9.; (not induced with TNF-α and IL-1β, but treated with lapatinib 25, 50, 100, and 200 μg/L doses), Flask 4., 6., 8., 10; after stimulation with TNF-α and IL-1β, 25, 50, 100, and 200 μg/l doses of lapatinib were added (Figure 1).

### 2.1. Biopsy collection and human synovial fibroblasts isolation and culture

The synovial tissue specimen was taken from a RA patient in remission at the age of 55, who underwent surgical joint replacement, and did not take immunosuppressive medication for 15 days. The RA patient fulfilled the American College of Rheumatology (ACR)/the European League Against Rheumatism (EULAR) 2010 classification criteria for RA.

The cartilage tissues were collected in phosphate buffer salt (PBS) with antibiotics and kept at 4 °C. The cartilage slices were minced and digested with 1% trypsin for 0.5 h at 37 °C and subsequently with 0.25% collagenase overnight in serum-free medium at 37 °C. After digestion, the suspension containing isolated synovial fibroblasts was centrifuged (1000 **×** g for 10 min). The resulting pellet was washed twice with Dulbecco’s modified eagles medium (DMEM) to remove the remaining digestive enzyme. Then, cells were resuspended and cultured in DMEM with 10% fetal bovine serum (FBS) and 1% antibiotic (Lonza, Switzerland). Within 2–3 days of harvesting, primary synovial fibroblasts were cultured to 80% confluence and plated in 24 well and 96-well plates [14].

### 2.2. In vitro inflammation study and lapatinib application in primary synovial fibroblasts culture

In this study, synovial fibroblasts were preexposed to proinflammatory cytokines such as IL-1β and TNF-α and initiated inflammation in synovial fibroblasts. Primary synovial fibroblasts cultures were incubated with serum-starved medium (0.5% FBS). Serum-starved human articular synovial fibroblasts were prestimulated with 5ng/ml IL-1β and 10 ng/ml TNF-α for 24 h before being treated with lapatinib (Glaxosmithkline San. ve Tic. AŞ, İstanbul, Turkey). After overnight, various concentrations (25, 50, 100, and 200 μM) of lapatinib were administered to inflammatory cells and incubated for 24 h [15].

### 2.3. ELISA tests

To investigate the effect of lapatinib on inflammatory cells, the levels of MMP-1, -3, and, -13 in the culture supernatant were measured using commercially available enzyme-linked immunosorbent assay (ELISA) kits. The samples were performed six times (n = 6).

### 2.4. MTS assay

For the cell proliferation assay, synovial fibroblasts were seeded in triplicate in 96 well plates for 24 h, and then cells were treated with 5 ng/mL IL-1β and 10 ng/mL TNF-α for 24 h. Then, various concentrations (25, 50, 100, and 200 μM) of lapatinib were administered to inflammatory cells and incubated.

### 2.5. Statistical analysis

Based on a previous study [16], the sample size of this study was eight per group and it was calculated based on an alpha error of 0.05 and a power of 80% and 90% (GPower3.1). Data were presented as median (min-max). Kruskal Wallis one-way analysis of variance was used for comparisons among the groups (percent viability, MMP-1, MMP-3, MMP-13), and the Mann-Whitney U test was used for dual comparisons (percent viability, MMP-1, MMP-3, MMP-13). Statistical evaluations were performed using the Statistical Package for the Social Sciences (SPSS) package program, version 14.0. A P value of < 0.05 was considered to be significant.

## 3. Results

To test the effect of EGFR blockade, 10 groups were created; Group 1 was the Control group, Group 2 was stimulated with TNF-α and IL-1β, Group 3 was added only lapatinib at doses of 25, 50, 100, 200 μg/L, and Group 4 was added lapatinib at doses of 25, 50, 100, 200 μg/L, after stimulating with TNF-α and IL-1β.

### 3.1. The productions of MMPs from synovial fibroblasts

**Figure 2:**
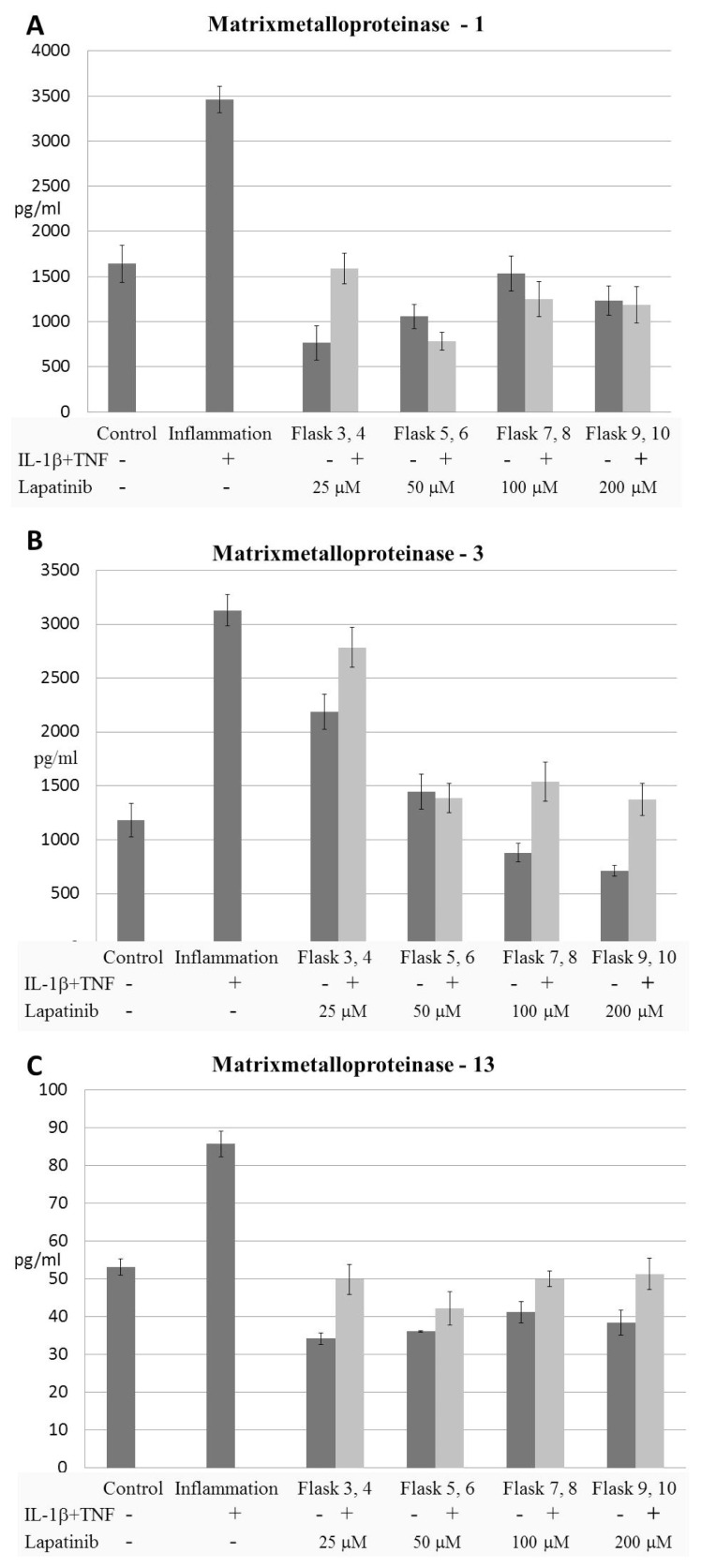
The productions of matrix metalloproteinases from synovial fibroblasts IL-1β and TNF-α applications increased the productions of MMP-1 (A), MMP-3 (B), and MMP-13 (C). Various concentrations of lapatinib decreased the productions of MMP-1 (A), MMP-3 (B), and MMP-13 (C) from RA-FLSs induced by IL-1β and TNF-α MMP; matrix metalloproteinase, IL; interleukin-1β, TNF-α; tumor necrosis factor.

The applications of IL-1β and TNF-α on synovial fibroblast culture increased the production of MMP-1,-3, and-13. On the other hand, lapatinib suppressed the productions of MMP-1,-3, and-13 from synovial fibroblasts induced by IL-1β and TNF-α (Table) (Figure 2A, 2B, 2C).

### 3.2. The proliferation of synovial fibroblasts

**Figure 3:**
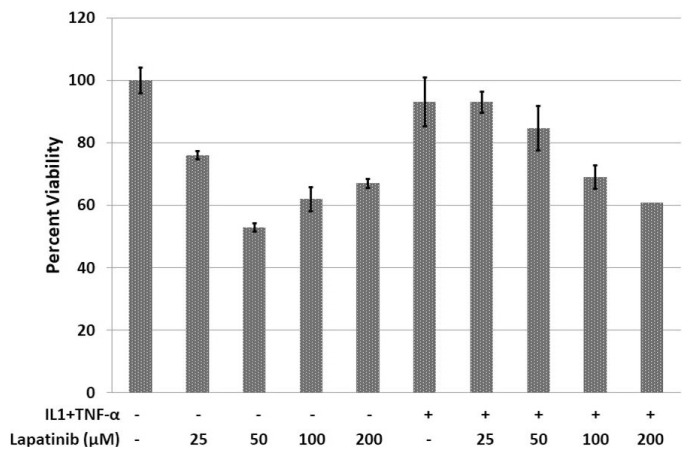
The viability of synovial fibroblasts. Various concentrations of lapatinib inhibited the proliferation of synovial fibroblasts. IL-1β; interleukin-1β, TNF-α; tumor necrosis factor-α

Synovial fibroblasts were isolated from the joint tissue obtained from the RA patient and stimulated with IL-1β and TNF-α to gain inflammatory character. The applications of IL-1β and TNF-α on synovial fibroblast cultures proliferated synovial fibroblasts (Figure 3). On the other hand, lapatinib suppressed the proliferation of synovial fibroblasts (Table) (Figure 2A, 2B, 2C).

## 4. Discussion

In this study, it was aimed to investigate the effectiveness of lapatinib, an EGFR tyrosine kinase inhibitor, on synovial fibroblasts. For this purpose, synovial fibroblast cell culture obtained from a patient with RA was stimulated with TNF-α and IL-1β cytokines. It was found that the level of matrix metalloproteinases increased after the stimulation. It was determined that when lapatinib was added to the medium (medium or cell culture) together with synovial fibroblasts TNF-α and IL-1β; MMP-1,-3, and -13 levels did not increase and their proliferation from synovial fibroblasts decreased.

The role of the EGF family and the EGFR signal pathway in angiogenesis regulation and hyperplastic growth of several tissues, as well as tumor growth, is well demonstrated [12,17,18]. Because synovial hyperplasia and pannus formation which invades cartilage and bone leading to joint destruction is similar to a tumor with its neovascularization and cellular infiltration, involvement of EGF and EGFR in RA pathology has been proposed [13,19,20].

In this study, it has been shown that cell proliferation is reduced when lapatinib, an EGFR inhibitor, is added to the synovial fibroblast cell culture. This result suggests that EGFR inhibition may prevent the proliferation of synovial fibroblasts, which are key effector cells in the destruction of the joint cartilage in RA.

Previous studies reported that there is EGFR expression in synovial tissue, and EGFR expression is increased in patients with RA compared to that in the healthy control group [21,22]. In this study, the suppression of MMP synthesis with lapatinib, an EGFR inhibitor, in the isolated synovial fibroblast cell culture, confirmed that synovial fibroblasts are sensitive to EGFR inhibition and synthesis EGFR.

As known, MMPs have collagenolytic activity. Indeed, tissue destruction of the extracellular matrix in RA is mediated by enzymatic cleavage predominantly by MMPs [23]. MMPs, cause tissue degradation in the synovial fibroblast microenvironment. Thus, they could make it easier for the synovium to invade the underlying tissues. There is a close link between radiological damage and the level of MMPs in RA [24]. In this study, the blockade of EGFR with lapatinib inhibited the synthesis of MMPs from synovial fibroblasts. This suggests that EGFR inhibition may prevent matrix degradation and joint damage in the treatment of RA.

MMPs are involved in angiogenesis. They lead to the loosening of endothelial tight junctions, reorganization of the actin cytoskeleton, and proteolysis of the basement membrane and interstitial matrix [25,26]. Moreover, it has been reported that MMPs stimulate the synthesis of angiogenic factors, degradation of antiangiogenic factors, and interactions with the cellular adhesion molecules expressed on the cell surface [27]. Conversely, when considering that EGF and EGFR have been demonstrated to induce the expressions of MMPs [28], our results predicted that blocking of EGFR may contribute to inhibition of angiogenesis in RA. In this study, MMP synthesis was suppressed by adding an EGFR inhibitor to the cell culture medium. These results indicate that EGFR inhibition may have a preventive role for pannus formation, for which angiogenesis is required.

There are some limitations to this study. The most important limitation is that our study is in vitro. In in vivo conditions, the effects of EGFR blockade may be different due to other mechanisms. It would have been better if lapatinib had also been tested in an animal model. In addition, the effect of EGFR blockade on inflammatory cytokine synthesis could also have been better.

In conclusion, synovial fibroblasts are sensitive to lapatinib, an EGFR inhibitor, and lapatinib suppresses MMP release from synovial fibroblasts. The significant role of MMP in the steps such as matrix degradation and angiogenesis, which have a major role in the pathogenesis of RA, suggests that the EGFR pathway and lapatinib may be promising pathways and agents in RA.

## Figures and Tables

**Table:** Matrix metalloproteinase -1, -3, and, -13 levels in the study groups.

		MMP-1 (pg/mL)	MMP-3 (pg/mL)	MMP-13 (pg/m)
**Control group**		1680 (1490–1870)^a^	1199 (891–1348)^a^	55.2 (49.0–60.3)^a^
**The group induced by TNF-α and IL-1β**		3440 (3245–3745)	3111 (2218–4268)	88.6 (74.2–94.2)
**Lapatinib 25 (μM)**	769 (625–980)^a^	2180 (1875–1281)^a^	32.2 (33.4–6.0)^a^
**Lapatinib 25 (μM) + TNF-α and IL-1β**	1591 (1165–1865)^a^	2790 (2045–4151)^a^	48.0 (36.4–71.6)^a^
**Lapatinib 50 (μM)**	1048 (905–1325)^a^	1460 (1228–1735)^a^	35.1 (35.9–36.4)^a^
**Lapatinib 50 (μM) + TNF-α and IL-1β**	779 (670–845)^a^	1398 (1268–1531)^a^	42.2 (38.1–46.8)^a^
**Lapatinib 100 (μM)**	1525 (1310–1670)^a^	860 (545–1141)^a^	41.2 (33.8–47.3)^a^
**Lapatinib 100 (μM) + TNF-α and IL-1β**	1242 (1030–1585)^a^	1524 (1291–1841)^a^	50.0 (47.7–51.6)^a^
**Lapatinib 200 (μM)**	1234 (930–1395)^a^	710 (561–861)^a^	38.4 (34.6–40.7)^a^
**Lapatinib 200 (μM) + TNF-α and IL-1β**	1194 (730–1475)^a^	1382 (921–2115)^a^	51.3 (48.1–5 ± 4)^a^

compared with the group induced by TNF-α and IL-1β, p < 0.05

MMP; Matrix metalloproteinase, TNF-α; Tumor necrosis factor-α, IL-1β; Interleukin-1β
